# Plasma Proteomic Landscape of Multidimensional Sleep Health and Its Association With Depression Risk Under Genetic Susceptibility

**DOI:** 10.1155/da/9191032

**Published:** 2026-10-09

**Authors:** Feng Chen, Yanggang Hong, Yue Zhu, Yukai Wang, Qi Chen, Jiajun Li, Yangguang Lu, Haoxiong Guan, Huixiang Sheng, Ruijie Xie, Fan Ye

**Affiliations:** ^1^ The Second School of Medicine Wenzhou Medical University Wenzhou Zhejiang China wmu.edu.cn; ^2^ The First Affiliated Hospital of Wenzhou Medical University Wenzhou China wzhospital.cn; ^3^ Public Health College Wenzhou Medical University Wenzhou Zhejiang China wmu.edu.cn; ^4^ School of Public Health Zhejiang University School of Medicine Hangzhou Zhejiang China zju.edu.cn; ^5^ The First School of Medicine School of Information and Engineering Wenzhou Medical University Wenzhou Zhejiang China wmu.edu.cn; ^6^ Faculty of Medicine Heidelberg University Heidelberg 69115 Germany heidelberg.edu; ^7^ Wenzhou Medical University Wenzhou Zhejiang China wmu.edu.cn

**Keywords:** depression, mediation analysis, multidimensional sleep health, plasma proteomics, polygenic risk score

## Abstract

**Background:**

Multidimensional sleep health, encompassing duration, snoring, chronotype, insomnia symptoms, and daytime sleepiness, is associated with various chronic diseases. However, its relationship with depression risk, especially the underlying molecular mechanisms across different levels of genetic susceptibility, remains unclear.

**Methods:**

In this prospective cohort study, we analyzed data from 36,852 UK Biobank participants, integrating multidimensional sleep scores, plasma proteomic data covering 2,911 proteins, and polygenic risk scores (PRS) for depression. Sleep‐related proteins were identified via elastic net regression, and their associations with incident depression were assessed using Cox proportional hazards models. Mediation and interaction analyses were conducted to explore the role of proteomic features and their effect modification by genetic risk.

**Results:**

Seventy‐three plasma proteins were selected by elastic net as sleep‐related proteomic features, mainly involving immune regulation, neural signaling, metabolic homeostasis, and extracellular matrix remodeling. The proteomic score derived from these features was positively correlated with sleep health (*r* = 0.234, *p* < 1*×*10^−100^). Healthy sleepers had a lower risk of depression (HR = 0.553, 95% CI: 0.454–0.674, *p* < 0.001), as did those with higher proteomic scores (HR = 0.660, 95% CI: 0.566–0.770, *p* < 0.001). Mediation analysis suggested that the proteomic signature statistically accounted for a modest proportion of the observed association between sleep health and incident depression. A significant additive interaction was observed between unhealthy sleep and high genetic risk (RERI = 0.280, 95% CI: 0.022–0.538).

**Conclusion:**

This study characterizes the plasma proteomic profile of multidimensional sleep health and identifies sleep‐associated proteomic signatures related to incident depression across levels of genetic susceptibility. These findings highlight potential biological pathways for further investigation and require independent replication and causal validation before clinical interpretation.

## 1. Introduction

Depression is a common and disabling mental disorder that affects more than 300 million people worldwide and represents one of the leading causes of global disease burden [1, 2]. Its etiology is multifactorial, involving the interplay of genetic susceptibility, environmental exposures, and psychosocial factors. In recent years, findings from genome‐wide association studies (GWAS) have demonstrated that depression has a strong polygenic basis, with multiple common genetic variants significantly associated with its development [3, 4]. However, the penetrance of these genetic factors is incomplete, suggesting that modifiable behavioral or biological factors may interact with genetic predisposition to influence depression risk.

Among these modifiable factors, sleep health has been widely recognized as a key determinant of both mental and physical well‐being. Sleep disturbances are closely related to the onset, recurrence, and treatment response to depression [5, 6]. Traditional research has often focused on individual sleep parameters, such as sleep duration or insomnia symptoms. Recently, the concept of multidimensional sleep health, which integrates multiple dimensions including duration, efficiency, rhythm, timing, and chronotype, has received increasing attention. This composite measure offers a more comprehensive assessment of overall sleep status [7] and has been linked to cardiovascular disease, metabolic syndrome, and all‐cause mortality [8]. However, its association with mental disorders, particularly depression, has not been systematically investigated, and the biological mechanisms underlying this relationship remain largely unexplored.

Plasma proteomics provides a systems‐level view of circulating proteins that reflect diverse biological processes (BPs), including immune and inflammatory responses, metabolic regulation, vascular function, and neurobiological changes. Large‐scale proteomic studies have demonstrated that circulating protein profiles capture age‐ and disease‐related biological variation and provide complementary information on systemic and neurological processes [9, 10]. In the field of mental health, a prospective analysis of 46,165 UK Biobank participants identified 157 baseline plasma proteins associated with incident depression, with prominent involvement of immune‐related pathways and associations with depression‐relevant brain structures and stress‐related exposures [11]. More recent proteomic profiling has further identified partially shared circulating protein signatures across depression and anxiety, including GDF15, PLAUR, and TNFRSF10B [12]. Beyond psychiatric disorders, plasma proteomics has also shown substantial relevance to cognitive and neurodegenerative outcomes. Prospective studies have identified proteins such as GFAP, NEFL, GDF15, and LTBP2 as markers associated with subsequent dementia [13], while independent cohort evidence has further demonstrated plasma proteomic signatures associated with cognitive decline and incident dementia [14]. Collectively, these findings suggest that circulating proteomic profiles capture BPs relevant to both psychiatric and neurodegenerative phenotypes and provide a useful molecular layer for investigating the biological correlates of modifiable behavioral factors such as sleep.

Circulating proteins represent a biologically plausible molecular layer through which sleep health may be linked to depression. Sleep is closely associated with systemic immune‐inflammatory activity, neuroendocrine and stress responses, metabolic regulation, and cellular signaling, many of which are reflected in circulating protein profiles [15, 16]. In turn, peripheral inflammatory and other protein‐mediated signals may communicate with the central nervous system through humoral, endothelial, neural, and immune pathways, potentially influencing neuroendocrine regulation, neurotransmission, and neuroplasticity relevant to depression. Importantly, such peripheral‐to‐central communication does not require individual plasma proteins to directly cross the blood–brain barrier because circulating signals may also influence brain function indirectly through endothelial activation, afferent neural pathways, and downstream immune signaling. These biological links provide a rationale for considering sleep‐associated plasma proteins as candidate biological intermediates in the sleep–depression association. However, because sleep characteristics and plasma proteins were assessed at the same baseline examination in the present study, our mediation analyses should be interpreted as statistical assessments of potential intermediate pathways rather than evidence of temporal or causal mediation.

Despite these advances, several important gaps remain. Although individual sleep characteristics and composite sleep health measures have been associated with depression, the circulating proteomic profile corresponding to multidimensional sleep health remains insufficiently characterized. It also remains unclear whether sleep‐associated proteomic features are prospectively associated with incident depression and to what extent they statistically account for the sleep–depression association. Moreover, although genetic susceptibility is an important determinant of depression, sleep health, circulating proteomic profiles, and polygenic risk have rarely been evaluated within a unified analytical framework. In particular, whether the associations of sleep health and sleep‐related proteomic signatures with depression differ across levels of genetic susceptibility and whether sleep health and genetic risk interact in relation to depression risk remain incompletely understood. Addressing these gaps requires an integrated framework that considers behavioral, molecular, and genetic dimensions together rather than examining each component in isolation.

In this study, we leveraged the UK Biobank to integrate multidimensional sleep health, plasma proteomic profiles, and polygenic risk for depression within a unified prospective framework. We aimed to characterize plasma proteomic features associated with multidimensional sleep health and construct a corresponding proteomic score, examine the prospective associations of sleep health and sleep‐associated proteomic features with incident depression and quantify the statistical contribution of the proteomic profile to the sleep–depression association, and further evaluate whether these associations differ across levels of genetic susceptibility and whether sleep health interacts with depression polygenic risk scores (PRS). By integrating behavioral, molecular, and genetic information, this study seeks to provide a more comprehensive characterization of the biological and genetic context underlying the association between multidimensional sleep health and depression risk.

## 2. Methods

### 2.1. Study Population

This study was based on the UK Biobank, a large‐scale ongoing prospective cohort study [17]. Between 2006 and 2010, over 500,000 participants aged 37–73 years were recruited from 22 assessment centers across England, Scotland, and Wales. Comprehensive baseline data were collected, including sociodemographic characteristics, lifestyle factors, physical function, anthropometric measurements, and biological samples. Ethical approval was obtained from the North West Multi‐Centre Research Ethics Committee (MREC reference: 21/NW/0157), and all participants provided written informed consent.

A total of 52,998 participants with available plasma proteomic data were initially included. We excluded 9704 participants with missing data on sleep‐related variables. To reduce population stratification bias in the PRS analysis, 2638 participants of non‐White ancestry were excluded, along with 179 individuals with missing depression PRS data. Furthermore, to minimize reverse causation, 3625 individuals with depression at baseline were removed based on self‐reports, electronic health records, and hospital admission data. The final analytical sample consisted of 36,852 participants. Details of the exclusion process are shown in Figure S1.

### 2.2. Assessment of Multidimensional Sleep Health

Sleep behavior data were obtained from baseline touchscreen questionnaires (http://biobank.ndph.ox.ac.uk/showcase/label.cgi?id=100057). Based on previous studies [7, 18], we included five key sleep traits: chronotype, sleep duration, insomnia symptoms, snoring, and daytime sleepiness. Each low‐risk behavior was assigned one point: being a morning or more morning‐type chronotype; sleeping 7–8 h per night; reporting never or rarely experiencing insomnia; absence of snoring; and low frequency of daytime sleepiness (“never/rarely” or “sometimes”). The total sleep score ranged from 0 to 5, with higher scores indicating healthier sleep patterns. Participants were categorized as having healthy (4–5 points), intermediate (2–3 points), or unhealthy (0–1 points) sleep patterns [19].

### 2.3. Depression Ascertainment

Incident depression was ascertained from linked hospital inpatient records using the International Classification of Diseases, 10th Revision (ICD‐10), specifically codes F32 (depressive episode) and F33 (recurrent depressive disorder). A participant was considered to have developed incident depression if either a primary or secondary hospital diagnosis contained one of these codes after baseline. Participants with evidence of depression before or at baseline were excluded, as described in the Study Population section. Follow‐up time was calculated from the date of baseline assessment to the earliest occurrence of incident depression, death, loss to follow‐up, or the administrative end of follow‐up on October 31, 2022, whichever occurred first.

### 2.4. Proteomic Profiling

Plasma proteomic profiling was performed using the Olink Explore 3072 platform on EDTA plasma samples collected at baseline from ~50,000 participants. The analytical procedures were detailed in previous publications [9]. Proteins were measured across eight panels, covering cardiometabolic (I and II), inflammation (I and II), neurology (I and II), and oncology (I and II), yielding 2923 unique proteins. After excluding 12 proteins with over 20% missing values, 2911 proteins were retained for analysis [10]. Missing measurements among the retained proteins were imputed using the protein‐specific cohort mean to maintain a complete proteomic matrix and minimize additional loss of observations, consistent with approaches used in previous large‐scale UK Biobank Olink proteomic analyses [9]. All protein levels were expressed as normalized protein expression (NPX), log_2_‐transformed, and standardized prior to analysis.

### 2.5. Polygenic Risk Score for Depression

The PRS for depression was based on the ICD‐10‐defined depression score developed by Cai et al. [20], comprising 21,510 variants. In the source study, PRSs were generated using the RICOPILI pipeline. Single nucleotide polymorphisms (SNPs) were selected using LD clumping and *p*‐value thresholding, with an LD threshold of *r* ^2^ < 0.1 and a GWAS *p*‐value threshold ≤0.1. Published variant weights were applied to imputed UK Biobank genotype data after alignment for effect allele direction. The PRS was calculated as the weighted sum of effect alleles (0, 1, or 2) multiplied by corresponding effect sizes. Variants were filtered by INFO ≥0.8, MAF ≥1%, and missingness ≤2%, and ambiguous SNPs (A/T or C/G) were excluded. Scores were standardized (*z*‐scores) and categorized into low (bottom 25%), intermediate (middle 50%), and high (top 25%) risk groups. Genetic principal components, genotyping array, and batch variables were not additionally included in the downstream regression models.

### 2.6. Covariates

Covariates included age, sex, educational attainment, Townsend deprivation index, body mass index (BMI), smoking and alcohol status, physical activity, history of cardiovascular disease, and diabetes. Education was categorized as college or above versus below college. The Townsend index assessed material deprivation based on postal codes and census data [21]. Smoking and drinking status were classified as never, former, or current. Physical activity was assessed using the International Physical Activity Questionnaire, focusing on time spent in moderate‐intensity activity. Self‐reported diagnoses or current use of relevant medications were used to identify cardiovascular disease and diabetes. Detailed UK Biobank field IDs and coding rules for selected covariates are provided in Table S3. Missing values in Townsend deprivation index, BMI, smoking status, alcohol status, moderate physical activity, diabetes, and cardiovascular disease were handled using multiple imputation by chained equations implemented in the mice R package [22]. The MICE procedure was run with five imputations and five iterations. Predictive mean matching was used for Townsend deprivation index and BMI, polytomous logistic regression for smoking and alcohol status, and logistic regression for moderate physical activity, diabetes, and cardiovascular disease. These seven covariates were jointly included in the imputation model.

### 2.7. Enrichment Analysis

To explore protein–protein interactions (PPIs), we constructed a PPI network using the STRING database (http://string-db.org), limited to *Homo sapiens* and a minimum confidence score of 0.40 [23]. Visualization and topological analysis were performed in Cytoscape (v3.10.2), with degree, closeness, and betweenness centrality calculated [24]. Gene Ontology (GO) and Kyoto Encyclopedia of Genes and Genomes (KEGG) enrichment analyses were performed on the Metascape platform (http://metascape.org). GO terms covered BP, molecular function (MF), and cellular component (CC). Criteria for significant enrichment included at least three overlapping genes, adjusted *p*‐value <0.05, and enrichment score >1.5.

### 2.8. Statistical Analysis

To identify sleep‐associated proteomic features, we applied elastic‐net regularized linear regression [25, 26] using the glmnet package, with the continuous multidimensional sleep health score (0–5) as the outcome and standardized plasma protein levels as predictors. The continuous score was used to preserve information across the full multidimensional sleep‐health gradient rather than reducing the score to binary or three‐category classifications. The categorical sleep patterns were retained separately for subsequent Cox regression analyses of incident depression. The primary analysis used *α* = 0.5 to balance L1 (Lasso) and L2 (Ridge) penalties, with 10‐fold cross‐validation used to determine the regularization parameter *λ*. The 1‐SE rule (*λ*_1SE_) was adopted for the primary model to favor a more parsimonious solution. The model was subsequently fitted at the selected *λ*, and proteins with nonzero coefficients were retained as sleep‐related proteomic features. For each participant, the proteomic score was calculated as the weighted sum of the standardized levels of the 73 selected proteins using their corresponding elastic‐net coefficients, excluding the intercept: $\text{Proteomic}\;{\text{score}}_{i}=\sum_{j=1}^{73}\beta_{j}X_{ij}$, where *X*_*ij*_ denotes the standardized plasma level of protein *j* for participant *i*, and *β_j_* denotes its corresponding elastic‐net coefficient. To evaluate the robustness of feature selection, we additionally examined *α* values of 0.10, 0.25, 0.50, 0.75, and 1.00 and compared *λ*_1SE_ with *λ*_min_, corresponding to the minimum cross‐validation error. We further repeated 10‐fold cross‐validation 50 times at *α* = 0.50 using different random fold assignments. Stability was evaluated using the proportion of primary‐model proteins reselected, Jaccard similarity between selected feature sets, selection frequencies of individual proteins, and Pearson correlations between the proteomic scores derived from alternative models and the primary proteomic score.

We calculated Pearson correlations between the proteomic score and sleep scores as well as between individual proteins and sleep scores. Distributions were assessed using the Kolmogorov–Smirnov test. Continuous variables were summarized as mean ± SD or median (IQR) and categorical variables as percentages. Group comparisons used *t*‐tests, nonparametric tests, or chi‐square tests as appropriate. Cox proportional hazards models were used to assess the effects of sleep patterns, proteomic scores, and PRS on depression risk, adjusting for covariates. For analyses treating the proteomic score as a continuous variable, hazard ratios were estimated per one‐unit increase in the original weighted proteomic score, which was not further standardized after construction. The proportional hazard assumption was tested using Schoenfeld residuals. Stratified analyses were conducted by PRS levels, and interaction terms were tested. Participants with healthy sleep and low genetic risk served as the reference group. Additive interaction was assessed using the relative excess risk due to interaction (RERI) and attributable proportion due to interaction (AP), calculated as RERI = HR_11_ − HR_10_ − HR_01_ + 1 and AP = RERI/HR_11_, respectively. Mediation analysis was performed using a product‐of‐coefficients approach to evaluate the extent to which the proteomic signature statistically accounted for the association between sleep pattern and incident depression. Unhealthy sleep served as the reference category, and intermediate and healthy sleep patterns were evaluated separately. For each comparison, a multivariable linear regression model was first fitted with the continuous proteomic score as the dependent variable to estimate the association between sleep pattern and the mediator (path *a*). A Cox proportional hazards model including both sleep pattern and the proteomic score was then fitted for incident depression to estimate the association between the mediator and the outcome (path *b*) and the direct association between sleep pattern and depression (path *c*′). A corresponding Cox model without the proteomic score was used to estimate the total association (path *c*). The indirect effect was calculated on the log‐hazard scale as *a* × *b* and exponentiated to obtain the corresponding indirect‐effect HR. The proportion mediated was calculated as (*a* × *b*)/*c* × 100%. Statistical uncertainty in the mediation proportion was assessed using 1000 nonparametric bootstrap resamples, from which percentile‐based 95% confidence intervals were obtained. All models were adjusted for age, sex, educational attainment, Townsend deprivation index, BMI, smoking status, alcohol status, moderate physical activity, cardiovascular disease, and diabetes. Because sleep characteristics and plasma proteins were measured at the same baseline assessment, the mediation analysis was interpreted as a statistical decomposition of the observed association rather than evidence of a causal mediation pathway.

Additional analyses included separate evaluations of five individual sleep traits using Cox models and sensitivity analysis excluding participants who developed depression within the first year of follow‐up. All analyses were conducted in R version 4.3.0, with two‐sided *p*‐values <0.05 considered statistically significant.

## 3. Results

### 3.1. Baseline Characteristics of Participants

A total of 36,852 participants were included in this study. During a median follow‐up of 13.72 years, 1834 participants developed incident depression. Baseline characteristics stratified by depression status are presented in Table 1. The overall mean age was 57.16 ± 8.14 years. Compared with participants who did not develop depression, those who did were less likely to be male (39.53% vs. 46.77%) and to have received higher education (41.17% vs. 47.95%), but had a higher BMI (28.37 ± 5.22 vs. 27.26 ± 4.65 kg/m^2^) and a higher Townsend deprivation index (median: –1.81 [IQR –3.53, 1.19] vs. –2.34 [–3.75, 0.12]); all differences were statistically significant (*p* < 0.001).

**Table 1:** Characteristics of participants classified by depression.

Characteristics	Total (*n* = 36,852)	Depression (*n* = 1834)		
		Yes	No	*p*‐Value
Age (years)	57.16 ± 8.14	57.09 ± 8.47	57.16 ± 8.12	0.731
Sex, *n* (%)				<0.001
Male	17,104 (46.41)	725 (39.53)	16,379 (46.77)	
Female	19,748 (53.59)	1109 (60.47)	18,639 (53.23)	
Education level, *n* (%)				<0.001
College or above	17,547 (47.61)	755 (41.17)	16,792 (47.95)	
Below college	19,305 (52.39)	1079 (58.83)	18,226 (52.05)	
Townsend deprivation index	−2.32 (−3.74, 0.17)	−1.81 (−3.53, 1.19)	−2.34 (−3.75, 0.12)	<0.001
BMI (kg/m^2^)	27.32 ± 4.69	28.37 ± 5.22	27.26 ± 4.65	<0.001
Smoking status, *n* (%)				<0.001
Never	19,820 (53.78)	847 (46.18)	18,973 (54.18)	
Former	13,401 (36.36)	726 (39.59)	12,675 (36.20)	
Current	3631 (9.85)	261 (14.23)	3370 (9.62)	
Alcohol status, *n* (%)				<0.001
Never	1126 (3.06)	86 (4.69)	1040 (2.97)	
Former	1204 (3.27)	110 (6.00)	1094 (3.12)	
Current	34,522 (93.68)	1638 (89.31)	32,884 (93.91)	
Moderate physical activity, *n* (%)				0.612
Yes	16,387 (44.47)	805 (43.89)	15,582 (44.50)	
No	20,465 (55.53)	1029 (56.11)	19,436 (55.50)	
Diabetes, *n* (%)				<0.001
Yes	1841 (5.00)	148 (8.07)	1693 (4.83)	
No	35,011 (95.00)	1686 (91.93)	33,325 (95.17)	
CVD, *n* (%)				<0.001
Yes	11,345 (30.79)	706 (38.50)	10,639 (30.38)	
No	25,507 (69.21)	1128 (61.50)	24,379 (69.62)	
Depression PRS, *n* (%)				<0.001
Low	9213 (25.00)	333 (18.16)	8880 (25.36)	
Intermediate	18,426 (50.00)	777 (42.37)	17,649 (50.40)	
High	9213 (25.00)	724 (39.48)	8489 (24.24)	
Sleep pattern, *n* (%)				<0.001
Unhealthy sleep pattern	1529 (4.15)	126 (6.87)	1403 (4.01)	
Intermediate sleep pattern	21,259 (57.69)	1166 (63.58)	20,093 (57.38)	
Healthy sleep pattern	14,064 (38.16)	542 (29.55)	13,522 (38.61)	

*Note:* Values are presented as mean ± standard deviation (SD) for continuous variables and number (percentage) for categorical variables, except for the Townsend deprivation index, which is presented as median (interquartile range). *p*‐values were calculated using Student’s *t*‐test or nonparametric tests, as appropriate, for continuous variables and chi‐square tests for categorical variables, comparing participants with and without depression. Depression PRS was categorized as low (bottom 25%), intermediate (middle 50%), and high (top 25%). Sleep pattern groups were categorized according to the predefined multidimensional sleep scoring algorithm.

Abbreviations: BMI, body mass index; CVD, cardiovascular disease; PRS, polygenic risk score.

Regarding smoking behavior, the depression group had a higher proportion of both former and current smokers (39.59% and 14.23% vs. 36.20% and 9.62%, respectively; *p* < 0.001). In terms of alcohol consumption, participants with depression were more likely to be never or former drinkers and less likely to be current drinkers (4.69%, 6.00%, and 89.31% vs. 2.97%, 3.12%, and 93.91%, respectively; *p* < 0.001). The proportion engaging in moderate‐intensity physical activity was similar between groups (43.89% vs. 44.50%, *p* = 0.612).

For comorbidities, participants with depression had higher rates of diabetes (8.07% vs. 4.83%) and cardiovascular disease (38.50% vs. 30.38%) (both *p* < 0.001). Genetically, the depression group had a higher proportion of individuals with high PRS and a lower proportion with low PRS (high: 39.48% vs. 24.24% and low: 18.16% vs. 25.36%).

Regarding sleep behaviors, those with depression had a higher prevalence of unhealthy and intermediate sleep patterns, and a lower proportion of healthy sleep patterns (6.87%, 63.58%, and 29.55% vs. 4.01%, 57.38%, and 38.61%, respectively; *p* < 0.001).

### 3.2. Proteomic Signatures of Multidimensional Sleep Health Patterns

Using elastic‐net regression with 10‐fold cross‐validation, 73 plasma proteins had nonzero coefficients at *λ*_1SE_ and were retained as sleep‐related proteomic features (Figure 1A). Proteins with higher positive coefficients, suggestive of better sleep health, included integrin αM (ITGAM), matrix remodeling‐associated protein 8 (MXRA8), amylase *α*‐2A (AMY2A), a disintegrin and metalloproteinase with thrombospondin motifs 13 (ADAMTS13), and insulin‐like growth factor‐binding protein 3 (IGFBP3). In contrast, proteins with higher negative coefficients, potentially indicative of poorer sleep health, included ERBB2, serum amyloid P component (APCS), butyrylcholinesterase (BCHE), GPI‐anchored receptor GFRA1, and protease PRSS8.

**Figure 1:**
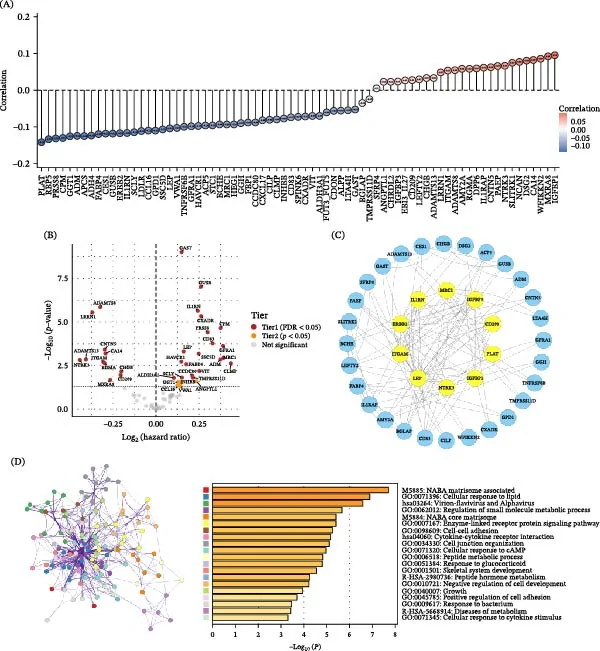
Proteomic landscape of multidimensional sleep health and depression risk. (A) Sleep health‐protein correlations for the 73 plasma proteins identified as sleep related. Points show Pearson correlation coefficients between each protein and the composite sleep health score; positive values indicate higher protein levels among participants with healthier sleep patterns. Proteins are annotated by gene symbols. (B) Associations of sleep‐related proteins with incident depression. The *x*‐axis shows log_2_ hazard ratios from Cox models and the *y*‐axis shows −log_10_ *p* values. Tier 1 denotes proteins significant after false discovery rate correction, Tier 2 denotes proteins significant at nominal level *p* < 0.05, and gray points are not significant. Selected proteins are labeled. Models were adjusted for age, sex, educational attainment, Townsend deprivation index, body mass index, smoking status, alcohol status, physical activity, cardiovascular disease, and diabetes. (C) Protein–protein interaction network of the 73 sleep‐related proteins. Node size reflects degree centrality and edge thickness reflects interaction confidence. Key hubs include LEP, ERBB2, NTRK3, ITGAM, IGFBP1, and IL1RN, linking immune, metabolic, and neurosignaling modules. (D) Functional enrichment of sleep‐related proteins. Bars display top Gene Ontology, KEGG, Reactome, and MSigDB matrisome terms ranked by −log_10_ *p*, highlighting cytokine–receptor interaction, cAMP‐mediated signaling, peptide hormone metabolism, responses to glucocorticoids and lipids, cell adhesion and junction organization, and core matrisome pathways. *Note:* Plasma proteins were quantified on the Olink Explore platform and normalized as NPX, log_2_ transformed, and standardized. Sleep‐related proteins were selected using elastic net with 10‐fold cross‐validation.

These proteins span a range of biological functions, including immune and inflammatory regulation such as ITGAM, ADM, and TNFRSF6B, extracellular matrix remodeling such as MXRA8 and ADAMTS13/8, neural signal transduction such as RGMA, ERBB2, and GFRA1, and metabolic regulation or protease activity such as PRSS8, GGT1, PLAT, and AMY2A. Pearson correlation analysis revealed a significant positive association between the proteomic score derived from these coefficients and the multidimensional sleep health score (*r* = 0.234, *p* < 1 × 10^−100^), indicating the biological relevance of this protein signature in reflecting sleep health status.

Robustness analyses supported the stability of the overall proteomic signature (Tables S1 and S2). When the *λ*_1SE_ rule was retained and α was varied from 0.10 to 1.00, 71–91 proteins were selected, with 97.3%–100.0% of the 73 primary proteins retained; the corresponding proteomic scores were highly correlated with the primary score (*r* = 0.999–1.000). Using *λ*_min_ resulted in larger feature sets of 392–406 proteins, while 66 of the 73 primary proteins (90.4%) were retained, and the resulting proteomic scores remained highly correlated with the primary score (*r* = 0.940–0.945). Across 50 repeated 10‐fold cross‐validation runs at *α* = 0.50 and *λ*_1SE_, the median number of selected proteins was 62 (IQR, 58–82), the median recovery rate of the primary 73‐protein set was 84.9% (IQR, 78.1%–100.0%), and the median Jaccard similarity with the primary feature set was 0.849 (IQR, 0.751–0.890). Of the 73 primary proteins, 39 were selected in all 50 repetitions, 48 were selected in at least 90% of repetitions, and 62 were selected in at least 70%. Despite variability in the exact membership of some individual features, the proteomic scores derived from the repeated cross‐validation models remained highly consistent with the primary score (median *r* = 0.999; IQR, 0.998–0.999).

### 3.3. Protein–Protein Interactions and Functional Enrichment of Sleep Health‐Associated Proteins

To explore the underlying functional mechanisms of the identified proteins, we constructed a protein–protein interaction network using the 73 sleep‐health‐related proteins and visualized it in Cytoscape (Figure 1C). The network showed a highly interconnected structure and identified several central hub proteins including LEP, ERBB2, NTRK3, ITGAM, IGFBP1, and IL1RN. These proteins exhibited high network centrality, suggesting that they may act as key regulators in sleep‐related molecular pathways by mediating interactions across immune‐inflammation, metabolic regulation, and cellular communication modules.

GO and KEGG enrichment analyses revealed that these proteins were significantly enriched in several BPs, including lipid response, cytokine‐receptor interaction, cell adhesion, cAMP‐mediated signaling, and peptide hormone metabolism (Figure 1D). Additional pathways involved enzyme‐linked receptor signaling, glucocorticoid responses, and skeletal system development. Many of these pathways are closely related to inflammation and metabolism, highlighting their potential roles in the biological link between sleep behavior and depression.

### 3.4. Identification of Depression‐Associated Proteins Within Sleep‐Related Signatures

We further examined the association between the sleep‐related proteomic features and depression risk (Figure 1B). Among the identified proteins, gastrin (GAST) and β‐glucuronidase (GUSB) showed the strongest positive associations with depression risk in fully adjusted Cox models (FDR <0.05), suggesting potential involvement in immune and metabolic mechanisms related to mood disorders. Other Tier 1 proteins that remained significant after FDR correction included ADAMTS8, IL1RN, GFRA1, LEP, PRSS8, CA14, MRC1, and ITGAM. These proteins participate in BPs such as cytokine regulation, cell adhesion, tissue remodeling, and lipid metabolism, highlighting their potential involvement in BPs relevant to both sleep health and depression. Additionally, several Tier 2 proteins including CNTN5, RGMA, CD83, ANGPTL1, CD209, CCL16, and GGT1 reached nominal significance (*p* < 0.05) and may warrant further investigation. Overall, these findings highlight a set of depression‐related proteins within the multidimensional sleep health signature that may warrant further investigation as candidate proteins associated with both sleep health and depression risk.

### 3.5. Associations of Sleep Health Patterns, Proteomic Signatures, and Genetic Risk With Depression

To assess the independent contributions of sleep health patterns, proteomic signatures, and genetic predisposition to depression risk, we fitted multivariable Cox proportional hazard models (Figure 2). Compared with unhealthy sleepers, individuals with intermediate sleep had a significantly lower risk of developing depression (HR = 0.740, 95% CI: 0.615–0.891, *p* = 0.001), and those with healthy sleep had a further lower risk (HR = 0.553, 95% CI: 0.454–0.674, *p* < 0.001), indicating a graded inverse association across sleep health categories.

**Figure 2:**
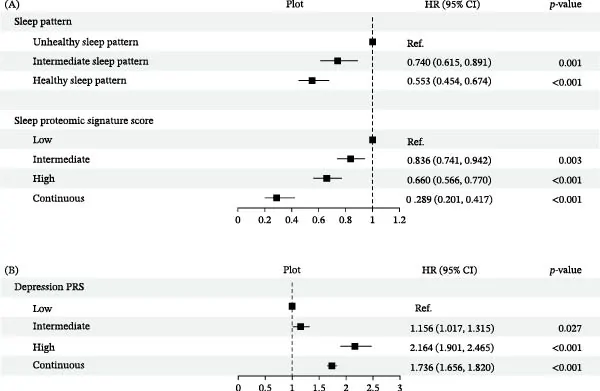
Associations of multidimensional sleep health, sleep‐related proteomic signature, and depression polygenic risk with incident depression. (A) Forest plot of hazard ratios for incident depression by sleep pattern and by tertiles of the sleep proteomic signature score, plus the score as a continuous variable, with the continuous estimate expressed per one‐unit increase in the original weighted proteomic score. Reference groups are unhealthy sleep for sleep pattern and low score for the proteomic signature. Points show multivariable Cox estimates and bars indicate 95% CI; *p* values are from Wald tests. (B) Forest plot of hazard ratios by categories of depression polygenic risk score and as a continuous variable, with low PRS as the reference. Models were adjusted for age, sex, educational attainment, Townsend deprivation index, body mass index, smoking status, alcohol status, physical activity, cardiovascular disease, and diabetes. HR < 1 indicates lower risk, HR > 1 indicates higher risk. CI, confidence interval; HR, hazard ratio; PRS, polygenic risk score.

The proteomic sleep score exhibited a similar trend. Compared with the low‐score group, individuals in the intermediate and high‐score groups had reduced risks of depression (HR = 0.836, 95% CI: 0.741–0.942, *p* = 0.003, and HR = 0.660, 95% CI: 0.566–0.770, *p* < 0.001, respectively). When analyzed continuously, each one‐unit increase in the original weighted proteomic score was associated with a lower risk of incident depression (HR = 0.289, 95% CI: 0.201–0.417, *p* < 0.001).

For genetic susceptibility, compared with the low PRS group, individuals in the intermediate and high PRS groups had increased depression risk (HR = 1.156, 95% CI: 1.017–1.315, *p* = 0.027, and HR = 2.164, 95% CI: 1.901–2.465, *p* < 0.001, respectively). Continuous PRS analysis confirmed a linear risk increase (HR = 1.736, 95% CI: 1.656–1.820, *p* < 0.001).

### 3.6. Mediation Analysis of Proteomic Features in the Sleep–Depression Association

Mediation analysis suggested that the proteomic signature statistically accounted for a modest proportion of the observed association between sleep pattern and incident depression (Figure S2). Among individuals with intermediate sleep patterns, the indirect effect through the proteomic score was HR = 0.975 (95% CI, 0.962–0.988), while the direct effect was HR = 0.765 (95% CI, 0.635–0.921), corresponding to a proportion mediated of 8.55% (95% CI, 3.69%–27.92%). Among individuals with healthy sleep patterns, the indirect effect was HR = 0.929 (95% CI, 0.898–0.961), and the direct effect was HR = 0.592 (95% CI, 0.482–0.728), corresponding to a proportion mediated of 12.23% (95% CI, 6.63%–21.67%). Because sleep characteristics and plasma proteins were measured concurrently at baseline, these findings should be interpreted as statistical mediation rather than evidence of a causal biological pathway.

### 3.7. Effect Modification by Genetic Susceptibility

Stratified analysis by depression PRS revealed heterogeneity in the associations of sleep patterns and proteomic scores with depression across genetic backgrounds (Figure 3). Using unhealthy sleep as the reference, an inverse association between healthy sleep and incident depression was observed across PRS strata. In the low PRS group, the association did not reach statistical significance (HR = 0.626, 95% CI: 0.388–1.008, *p* = 0.054), but it was significant in the intermediate PRS group (HR = 0.609, 95% CI: 0.445–0.834, *p* = 0.002) and the high PRS group (HR = 0.470, 95% CI: 0.347–0.637, *p* < 0.001). This trend suggests that the inverse association between healthy sleep and incident depression was more pronounced among individuals with higher genetic risk.

**Figure 3:**
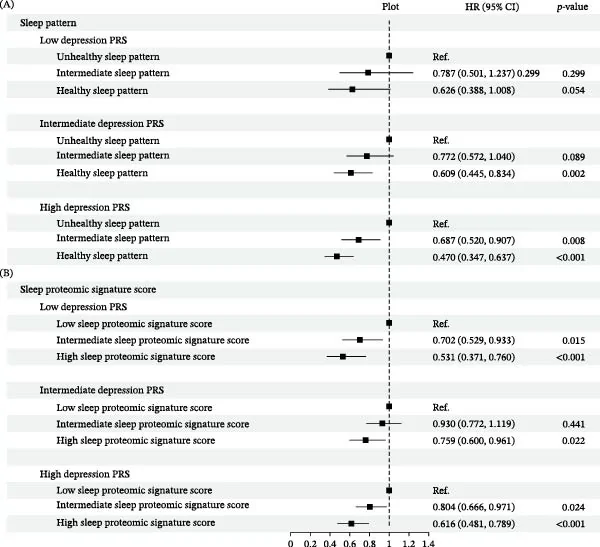
Associations of sleep health and the sleep proteomic signature with incident depression across levels of genetic susceptibility. (A) Associations between sleep pattern and incident depression stratified by depression PRS level. Within each PRS stratum, unhealthy sleep serves as the reference; points show adjusted hazard ratios with 95% confidence intervals and corresponding *p* values. (B) Associations between tertiles of the sleep proteomic signature score and incident depression stratified by PRS, with the low score as the reference in each stratum. Models were adjusted for the same covariates as in Figure 2. Inverse associations between higher sleep proteomic signature scores and incident depression were observed across PRS strata, although the magnitude of these associations did not show a clear monotonic pattern according to genetic risk. CI, confidence interval; HR, hazard ratio; PRS, polygenic risk score.

Similarly, higher proteomic scores were inversely associated with incident depression across all three PRS strata, although the magnitude of the associations did not show a clear monotonic pattern across increasing levels of genetic risk. Compared with the low proteomic‐score group, the HRs for the high proteomic‐score group were 0.531 (95% CI: 0.371–0.760) in the low PRS group, 0.759 (95% CI: 0.600–0.961) in the intermediate PRS group, and 0.616 (95% CI: 0.481–0.789) in the high PRS group. Interaction analysis indicated a significant additive interaction between unhealthy sleep and high genetic risk (RERI = 0.280, 95% CI: 0.022–0.538; AP = 0.144, 95% CI: 0.071–0.216), consistent with a positive additive interaction between unhealthy sleep and higher genetic susceptibility.

### 3.8. Contribution of Sleep Subcomponents and Sensitivity Analyses

To further investigate the contribution of individual components of the multidimensional sleep pattern, we conducted subdimension‐specific analyses (Figure S3). Abnormal sleep duration, defined as less than 7 h or more than 8 h, was associated with an increased risk of depression compared to the reference range of 7–8 h (HR = 1.440, 95% CI: 1.311–1.582, *p* < 0.001). Insomnia (HR = 1.576, 95% CI: 1.391–1.786, *p* < 0.001) and excessive daytime sleepiness (HR = 1.748, 95% CI: 1.413–2.163, *p* < 0.001) were also significantly associated with increased risk. In contrast, chronotype (*p* = 0.053) and snoring (*p* = 0.132) were not statistically significant. These findings suggest that specific components of sleep structure, particularly sleep duration, insomnia, and daytime sleepiness, may play key roles in the relationship between sleep health and depression.

We performed a sensitivity analysis to address potential reverse causality bias (Figure S4). After excluding participants diagnosed with depression during the first year of follow‐up, the associations among sleep patterns, proteomic scores, PRS, and depression risk remained robust. Specifically, intermediate and healthy sleep patterns continued to be associated with significantly lower depression risk (HR = 0.732 and 0.540, *p* = 0.001 and *p* < 0.001, respectively). High proteomic scores remained inversely associated with depression risk (HR = 0.658, 95% CI: 0.562–0.771, *p* < 0.001), and elevated PRS continued to confer increased risk (HR = 1.177 and 2.160, *p* = 0.015 and *p* < 0.001, respectively). Continuous variable analyses yielded consistent results, reinforcing the validity and reliability of the findings.

### 3.9. Performance Evaluation of Multi‐Omics‐Based Predictive Models

To systematically evaluate the incremental predictive value of multidimensional sleep‐related proteomic features and depression PRS, we developed four Cox regression models with stepwise inclusion of variables in the full cohort (Table 2). The base model (Model 1), which included only clinical covariates, achieved a C‐index of 0.620. When the proteomic score was added (Model 2), the C‐index increased to 0.627 (Δ*C* = 0.007, 95% CI: 0.003–0.012), along with significant improvements in reclassification metrics (NRI = 0.077 and IDI = 0.001).

**Table 2:** Comparative performance of four Cox models for predicting incident depression in UK Biobank participants.

Metrics	UK Biobank
Depression cases/total sample size	1834/36852
C‐index (Model 1)	0.620
C‐index (Model 2)	0.627
C‐index (Model 3)	0.653
C‐index (Model 4)	0.659
C‐index comparisons (Δ*C*, 95% CI)	
Model 1 vs. Model 2	0.007 (0.003, 0.012)
Model 1 vs. Model 3	0.033 (0.024, 0.044)
Model 1 vs. Model 4	0.039 (0.029, 0.050)
Model 2 vs. Model 4	0.032 (0.023, 0.042)
Model 3 vs. Model 4	0.005 (0.002, 0.009)
NRI	
Model 1 vs. Model 2	0.077 (0.046, 0.102)
Model 1 vs. Model 3	0.159 (0.132, 0.191)
Model 1 vs. Model 4	0.170 (0.139, 0.197)
Model 2 vs. Model 4	0.158 (0.130, 0.190)
Model 3 vs. Model 4	0.075 (0.044, 0.101)
IDI	
Model 1 vs. Model 2	0.001 (0.001, 0.002)
Model 1 vs. Model 3	0.029 (0.022, 0.039)
Model 1 vs. Model 4	0.031 (0.023, 0.041)
Model 2 vs. Model 4	0.030 (0.023, 0.041)
Model 3 vs. Model 4	0.002 (0.001, 0.003)

*Note:* Model 1 includes baseline clinical risk factors: Townsend index, Sex, BMI, Age, Smoking, Drinking, Moderate activity, Education level, Diabetes, and CVD. Model 2 adds sleep‐related proteomic signature score to Model 1. Model 3 adds depression PRS to Model 1. Model 4 includes all predictors in Model 1, plus sleep proteomic signature and depression PRS. The models were evaluated by Harrell’s C‐index, NRI, and IDI, using 1000 bootstrap replicates. Δ*C*‐index denotes difference in C‐indices between models, with 95% confidence intervals in parentheses.

Abbreviations: IDI, integrated discrimination improvement; NRI, net reclassification improvement; PRS, polygenic risk score.

Incorporating the depression PRS into the base model (Model 3) resulted in a more substantial increase in model discrimination (C‐index = 0.653; Δ*C* = 0.033, 95% CI: 0.024–0.044), with larger gains in reclassification (NRI = 0.159 and IDI = 0.029). The final model (Model 4), which included both the proteomic score and PRS, demonstrated the best performance (C‐index = 0.659), representing an improvement of Δ*C* = 0.039 compared to Model 1 and Δ*C* = 0.032 compared to Model 2. This model also achieved the highest values for NRI (0.170) and IDI (0.031), indicating incremental improvements in discrimination and reclassification.

These results suggest that proteomic features derived from multidimensional sleep health and genetic susceptibility to depression provide complementary and independent predictive information. When integrated, they offer enhanced ability to identify individuals at elevated risk for depression beyond traditional clinical factors.

## 4. Discussion

Leveraging the extensive prospective dataset of the UK Biobank, this study systematically characterized the plasma proteomic landscape associated with multidimensional sleep health patterns. It further examines the longitudinal associations between these proteomic features and subsequent risk of depression, as well as the potential moderating role of individual genetic susceptibility. We found that better multidimensional sleep health was significantly associated with a series of plasma proteins involved in neuroinflammatory responses, metabolic homeostasis, and cell signaling. Some of these proteins were prospectively linked to the risk of incident depression, suggesting their potential as biological markers. In addition, inverse associations between the sleep‐related proteomic score and incident depression were observed across PRS strata, although their magnitude did not show a clear monotonic pattern according to genetic risk. In contrast, the inverse association between healthy sleep and incident depression appeared more pronounced among individuals with higher genetic susceptibility. These findings deepen our understanding of the complex interactions between sleep behaviors, protein expression, and mental disorders.

Sleep behavior plays a critical role in regulating the function of the hypothalamic‐pituitary‐adrenal axis, alleviating systemic inflammation, maintaining neurotransmitter homeostasis, and stabilizing the neural circuits involved in emotion regulation [15, 16, 27]. In contrast, sleep disturbances may contribute to the onset and progression of depression through mechanisms such as chronic low‐grade inflammation, enhanced physiological stress responses, and impaired brain plasticity. In recent years, multiple population‐based studies have consistently demonstrated a stable inverse relationship between composite sleep health scores, which integrate dimensions such as sleep duration, efficiency, insomnia symptoms or wake latency, circadian preference, sleep timing, and the risk of depression. For instance, in a study of older women, each unit increase in the multidimensional sleep health score was significantly associated with a reduced likelihood of developing depressive symptoms in the future [28]. Wallace and colleagues replicated this association in multiple international cohorts, strengthening its generalizability [29]. Our study similarly observed a graded association, with the risk estimates for incident depression decreasing from the unhealthy sleep group to the intermediate and healthy sleep groups. Importantly, this association remained consistent across different levels of genetic risk, and the inverse association between healthy sleep and incident depression was particularly pronounced among individuals with high PRS. The interaction analysis suggested a positive additive interaction, with an AP of 0.144, indicating that ~14.4% of the joint effect among participants with both exposures was attributable to the additive interaction. These findings suggest that the association between sleep health and incident depression may differ according to genetic susceptibility. However, given the observational nature of the study, it should not be interpreted as evidence that healthy sleep causally offsets genetically conferred depression risk. Further studies are needed to determine whether these observed associations have implications for depression prevention.

Our proteomic analysis provides biological context for the observed association between multidimensional sleep health and depression risk. Sleep‐related proteins were broadly enriched in pathways related to immune and inflammatory responses, metabolic regulation, and cellular signaling. These pathways included cytokine‐receptor interactions, cAMP‐mediated signaling, peptide hormone metabolism, lipid and glucocorticoid responses, cell adhesion, and core matrisome components [30, 31]. The protein–protein interaction network demonstrated a multicentric structure, with hub proteins such as LEP, ERBB2, NTRK3, ITGAM, IGFBP1, and IL1RN. These hub proteins occupied integrative positions connecting immune, metabolic, and neurosignaling modules and may represent candidate markers of cross‐system BPs [32–35]. Further analyses showed that several proteins, including GAST and GUSB, were significantly associated with increased depression risk. Others such as ADAMTS8, IL1RN, GFRA1, LEP, PRSS8, CA14, MRC1, and ITGAM remained statistically significant after false discovery rate correction. Additional proteins, including CNTN5, RGMA, CD83, ANGPTL1, CD209, CCL16, and GGT1, displayed nominal associations in the same direction. Collectively, these findings suggest the involvement of a potential biological cascade characterized by immune activation, extracellular matrix remodeling, and the coupling of metabolic and receptor signaling [11, 36–38]. These proteomic features are consistent with the epidemiological associations observed in our population‐level analyses and highlight potential biological pathways linking sleep health with depression risk.

This study has several notable strengths. First, it is based on a large prospective population‐based cohort, allowing for long‐term tracking of multidimensional sleep health and depression incidence and providing prospective evidence for their association. Second, the integration of high‐throughput plasma proteomic data with pathway enrichment and protein interaction network analyses identified biological pathways potentially relevant to the observed association between sleep health and depression. Third, by considering the interaction between genetic susceptibility and sleep behavior, the study provides additional insight into the heterogeneity of these associations across levels of genetic risk. Nevertheless, several limitations should be acknowledged. The proteomic data were derived from peripheral plasma and may not fully reflect dynamic changes occurring in the central nervous system. The proteomic measurements were cross‐sectional, limiting our ability to examine longitudinal trajectories. Because sleep characteristics and plasma proteins were measured at the same baseline assessment, their temporal ordering cannot be established; therefore, the mediation analysis should be interpreted as a statistical assessment of a potential intermediate pathway rather than evidence of causal mediation. Although the analyses were restricted to participants of White ancestry, genetic principal components, genotyping array, and batch variables were not additionally included in the downstream regression models; therefore, residual population structure and technical variation cannot be completely excluded. In addition, because the source GWAS used to derive the depression PRS included a proportion of UK Biobank participants, potential overlap between the discovery and target samples may have led to some overestimation of PRS‐related associations. Although the composite proteomic signature showed high robustness across alternative penalty specifications and repeated cross‐validation, some individual proteins showed lower selection frequencies, indicating uncertainty in the exact membership of weaker features. Accordingly, the elastic‐net‐selected proteins should be interpreted primarily as components of a multivariable proteomic signature rather than as definitive standalone biomarkers, and their individual reproducibility requires confirmation in independent cohorts. Although we adjusted for a broad set of demographic, socioeconomic, lifestyle, and cardiometabolic covariates, residual confounding by unmeasured or incompletely captured psychiatric, psychosocial, and medical factors cannot be excluded. We did not additionally adjust for every comorbid condition potentially associated with depression because some of these factors may lie on, or partly reflect, pathways linking sleep health to depression, and indiscriminate adjustment could introduce overadjustment. In addition, certain psychosocial characteristics, such as marital status, were not directly available as comprehensive baseline measures for the full analytic cohort. Finally, the findings were primarily based on individuals of White ancestry, and further research is needed to determine their generalizability to other populations and cultural contexts.

## 5. Conclusions

Based on large‐scale prospective population data, this study identified associations between multidimensional sleep health patterns and incident depression and characterized circulating proteomic features associated with sleep health. Several sleep‐associated proteins were also associated with subsequent depression risk and were enriched in pathways involving immune‐inflammatory regulation, metabolic homeostasis, and cellular signaling, highlighting potential biological pathways for further investigation. These findings are associative and hypothesis‐generating. Replication in independent cohorts and causal validation are required before the identified proteomic signatures can inform clinical intervention or individualized prevention.

## Author Contributions

**Feng Chen**: conceptualization, formal analysis, data curation, funding acquisition, writing – original draft, writing – review and editing. **Yanggang Hong**: conceptualization, methodology, writing – original draft, writing – review and editing. **Yue Zhu**: conceptualization, writing – original draft, writing – review and editing. **Yukai Wang**: conceptualization, writing – original draft. **Qi Chen**: writing – original draft. **Jiajun Li**: writing – original draft. **Yangguang Lu**: writing – original draft. **Haoxiong Guan**: writing – original draft. **Huixiang Sheng**: writing – original draft. **Ruijie Xie**: writing – review and editing, supervision. **Fan Ye**: writing – review and editing, supervision.

## Funding

This work was supported by the National Innovation and Entrepreneurship Training Program for College Students (Grant 202410343059) and the Zhejiang Xinmiao College Students Innovation and Entrepreneurship Training Program, China (Grant 2025R413A039), both awarded to Feng Chen. This work was also supported by the Applied Basic Research Program of the Wenzhou Municipal Science and Technology Bureau (Project Number YC20250329).

## Disclosure

All authors have read the manuscript and approved its publication.

## Ethics Statement

This study was conducted in accordance with the principles outlined in the Declaration of Helsinki. UK Biobank has ethics approval from the North West Multi‐Centre Research Ethics Committee (21/NW/0157). Appropriate informed consent was obtained from participants, and ethical approval was covered by the UK Biobank. This research has been conducted using the UK Biobank Resource under project number 838449.

## Conflicts of Interest

The authors declare no conflicts of interest.

## Supporting Information

Additional supporting information can be found online in the Supporting Information section.

## Supporting information


**Supporting Information** Figure S1: Flowchart of study participant selection in the UK Biobank. Figure S2: Mediation analysis of the association between sleep pattern and incident depression through the proteomic signature. Figure S3: Association between individual sleep components and risk of incident depression. Figure S4: Sensitivity analysis excluding incident depression within the first year of follow‐up. Table S1: Sensitivity of elastic‐net feature selection across alternative *α* values and *λ*‐selection rules. Table S2: Selection stability of the 73 primary sleep‐related proteomic features across 50 repeated 10‐fold cross‐validation runs. Table S3: Definitions and coding of selected covariates used in the analysis.

## Data Availability

The UK Biobank data are available on application (www.ukbiobank.ac.uk/).

## References

[1] GBD 2019 Mental Disorders Collaborators , Global, Regional, and National Burden of 12 Mental Disorders in 204 Countries and Territories, 1990–2019: A Systematic Analysis for the Global Burden of Disease Study 2019, The Lancet Psychiatry. (2022) 9, no. 2, 137–150, 10.1016/S2215-0366(21)00395-3. PMC8776563 35026139

[2] GBD 2019 Diseases and Injuries Collaborators , Global Burden of 369 Diseases and Injuries in 204 Countries and Territories, 1990–2019: A Systematic Analysis for the Global Burden of Disease Study 2019, The Lancet. (2020) 396, no. 10258, 1204–1222, 10.1016/S0140-6736(20)30925-9. PMC7567026 33069326

[3] Wray N. R. , Ripke S. , and Mattheisen M. , et al.Genome-Wide Association Analyses Identify 44 Risk Variants and Refine the Genetic Architecture of Major Depression, Nature Genetics. (2018) 50, no. 5, 668–681, 10.1038/s41588-018-0090-3. PMC5934326 29700475

[4] Howard D. M. , Adams M. J. , and Clarke T. K. , et al.Genome-Wide Meta-Analysis of Depression Identifies 102 Independent Variants and Highlights the Importance of the Prefrontal Brain Regions, Nature Neuroscience. (2019) 22, no. 3, 343–352, 10.1038/s41593-018-0326-7. PMC6522363 30718901

[5] Fang Y. , Forger D. B. , Frank E. , Sen S. , and Goldstein C. , Day-to-Day Variability in Sleep Parameters and Depression Risk: A Prospective Cohort Study of Training Physicians, NPJ Digital Medicine. (2021) 4, no. 1, 10.1038/s41746-021-00400-z. PMC7892862 33603132

[6] Li L. , Wu C. , Gan Y. , Qu X. , and Lu Z. , Insomnia and the Risk of Depression: A Meta-Analysis of Prospective Cohort Studies, BMC Psychiatry. (2016) 16, no. 1, 10.1186/s12888-016-1075-3. PMC5097837 27816065

[7] Fan M. , Sun D. , and Zhou T. , et al.Sleep Patterns, Genetic Susceptibility, and Incident Cardiovascular Disease: A Prospective Study of 385 292 UK Biobank Participants, European Heart Journal. (2020) 41, no. 11, 1182–1189, 10.1093/eurheartj/ehz849. PMC7071844 31848595

[8] Chung J. , Goodman M. , and Huang T. , et al.Multi-Dimensional Sleep and Mortality: The Multi-Ethnic Study of Atherosclerosis, Sleep. (2023) 46, no. 9, 10.1093/sleep/zsad048, zsad048. PMC10848217 37523657

[9] Sun B. B. , Chiou J. , and Traylor M. , et al.Plasma Proteomic Associations With Genetics and Health in the UK Biobank, Nature. (2023) 622, no. 7982, 329–338, 10.1038/s41586-023-06592-6. PMC10567551 37794186

[10] Zhu K. , Li R. , and Yao P. , et al.Proteomic Signatures of Healthy Dietary Patterns are Associated With Lower Risks of Major Chronic Diseases and Mortality, Nature Food. (2025) 6, no. 1, 47–57, 10.1038/s43016-024-01059-x. 39333296

[11] Kang J. , Yang L. , and Jia T. , et al.Plasma Proteomics Identifies Proteins and Pathways Associated With Incident Depression in 46,165 Adults, Science Bulletin. (2025) 70, no. 4, 573–586, 10.1016/j.scib.2024.09.041. 39424455

[12] Ding C. , Qi W. , Tu H. , Wang Y. , Zhang T. , and Sun H. , Proteomic Profiling of Human Plasma for Anxiety and Depression: Discovery of Potential Biomarkers and Mechanistic Insights, Journal of Affective Disorders. (2025) 391, 10.1016/j.jad.2025.120067, 120067. 40816366

[13] Guo Y. , You J. , and Zhang Y. , et al.Plasma Proteomic Profiles Predict Future Dementia in Healthy Adults, Nature Aging. (2024) 4, no. 2, 247–260, 10.1038/s43587-023-00565-0. 38347190

[14] Sim M. A. , Doecke J. D. , and Liew O. W. , et al.Plasma Proteomics for Cognitive Decline and Dementia—A Southeast Asian Cohort Study, Alzheimer’s & Dementia: The Journal of the Alzheimer’s Association. (2025) 21, no. 2, 10.1002/alz.14577, e14577. PMC11854348 39998981

[15] Ballesio A. , Inflammatory Hypotheses of Sleep Disturbance-Depression Link: Update and Research Agenda, Brain, Behavior, & Immunity-Health. (2023) 31, 10.1016/j.bbih.2023.100647, 100647. PMC10319168 37408788

[16] Hassamal S. , Chronic Stress, Neuroinflammation, and Depression: An Overview of Pathophysiological Mechanisms and Emerging Anti-Inflammatories, Frontiers in Psychiatry. (2023) 14, 10.3389/fpsyt.2023.1130989, 1130989. PMC10213648 37252156

[17] Sudlow C. , Gallacher J. , and Allen N. , et al.UK Biobank: An Open Access Resource for Identifying the Causes of a Wide Range of Complex Diseases of Middle and Old Age, PLOS Medicine. (2015) 12, no. 3, 10.1371/journal.pmed.1001779, e1001779. PMC4380465 25826379

[18] Cong H. , Li Z. , and Zhang J. , et al.Adherence to a Healthy Sleep Pattern and the Risk of Kidney Stone Disease: A Prospective Study of UK Biobank, Journal of Psychosomatic Research. (2025) 189, 10.1016/j.jpsychores.2024.111999, 111999. 39644883

[19] Zhuang Z. , Dong X. , Jia J. , Liu Z. , Huang T. , and Qi L. , Sleep Patterns, Plasma Metabolome, and Risk of Incident Type 2 Diabetes Mellitus, The Journal of Clinical Endocrinology and Metabolism. (2023) 108, no. 10, e1034–e1043, 10.1210/clinem/dgad218. 37084357

[20] Cai N. , Revez J. A. , and Adams M. J. , et al.Minimal Phenotyping Yields Genome-Wide Association Signals of Low Specificity for Major Depression, Nature Genetics. (2020) 52, no. 4, 437–447, 10.1038/s41588-020-0594-5. PMC7906795 32231276

[21] Fry A. , Littlejohns T. J. , and Sudlow C. , et al.Comparison of Sociodemographic and Health-Related Characteristics of UK Biobank Participants With Those of the General Population, American Journal of Epidemiology. (2017) 186, no. 9, 1026–1034, 10.1093/aje/kwx246. PMC5860371 28641372

[22] van Buuren S. and Groothuis-Oudshoorn K. , Multivariate Imputation by Chained Equations in R, Journal of Statistical Software. (2011) 45, no. 3, 1–67, 10.18637/jss.v045.i03.

[23] Szklarczyk D. , Kirsch R. , and Koutrouli M. , et al.The STRING Database in 2023: Protein-Protein Association Networks and Functional Enrichment Analyses for Any Sequenced Genome of Interest, Nucleic Acids Research. (2023) 51, no. D1, D638–D646, 10.1093/nar/gkac1000. PMC9825434 36370105

[24] Shannon P. , Markiel A. , and Ozier O. , et al.Cytoscape: A Software Environment for Integrated Models of Biomolecular Interaction Networks, Genome Research. (2003) 13, no. 11, 2498–2504, 10.1101/gr.1239303. PMC403769 14597658

[25] Tian F. , Wang Y. , and Qian Z. M. , et al.Plasma Metabolomic Signature of Healthy Lifestyle, Structural Brain Reserve and Risk of Dementia, Brain: A Journal of Neurology. (2025) 148, no. 1, 143–153, 10.1093/brain/awae257. 39324695

[26] Friedman J. , Hastie T. , and Tibshirani R. , Regularization Paths for Generalized Linear Models via Coordinate Descent, Journal of Statistical Software. (2010) 33, no. 1, 1–22, 10.18637/jss.v033.i01. PMC2929880 20808728

[27] Fang H. , Tu S. , Sheng J. , and Shao A. , Depression in Sleep Disturbance: A Review on a Bidirectional Relationship, Mechanisms and Treatment, Journal of Cellular and Molecular Medicine. (2019) 23, no. 4, 2324–2332, 10.1111/jcmm.14170. PMC6433686 30734486

[28] Furihata R. , Hall M. H. , and Stone K. L. , et al.An Aggregate Measure of Sleep Health is Associated With Prevalent and Incident Clinically Significant Depression Symptoms Among Community-Dwelling Older Women, Sleep. (2017) 40, no. 3, 10.1093/sleep/zsw075, zsw075. PMC6084764 28364417

[29] Hoepel S. J. W. , Oryshkewych N. M. , and Barnes L. L. P. , et al.Sleep Health Profiles Across Six Population-Based Cohorts and Recommendations for Validating Clustering Models, Sleep Health. (2025) 11, no. 3, 371–381, 10.1016/j.sleh.2025.01.012. PMC12140883 40118730

[30] Blanco I. and Conant K. , Extracellular Matrix Remodeling With Stress and Depression: Studies in Human, Rodent and Zebrafish Models, The European Journal of Neuroscience. (2021) 53, no. 12, 3879–3888, 10.1111/ejn.14910. PMC7967247 32673433

[31] Göver T. and Slezak M. , Targeting Glucocorticoid Receptor Signaling Pathway for Treatment of Stress-Related Brain Disorders, Pharmacological Reports. (2024) 76, no. 6, 1333–1345, 10.1007/s43440-024-00654-w. PMC11582215 39361217

[32] Burrows K. , McNaughton B. A. , and Figueroa-Hall L. K. , et al.Elevated Serum Leptin is Associated With Attenuated Reward Anticipation in Major Depressive Disorder Independent of Peripheral C-Reactive Protein Levels, Scientific Reports. (2023) 13, no. 1, 10.1038/s41598-023-38410-4, 11313. PMC10344903 37443383

[33] Feng Y. , Vetró A. , and Kiss E. , et al.Association of the Neurotrophic Tyrosine Kinase Receptor 3 (NTRK3) Gene and Childhood-Onset Mood Disorders, The American Journal of Psychiatry. (2008) 165, no. 5, 610–616, 10.1176/appi.ajp.2007.07050805. 18347002

[34] Schmid M. C. , Khan S. Q. , and Kaneda M. M. , et al.Integrin CD11b Activation Drives Anti-Tumor Innate Immunity, Nature Communications. (2018) 9, no. 1, 10.1038/s41467-018-07387-4, 5379. PMC6300665 30568188

[35] Wang H. , He Y. , and Sun Z. , et al.Microglia in Depression: An Overview of Microglia in the Pathogenesis and Treatment of Depression, Journal of Neuroinflammation. (2022) 19, no. 1, 10.1186/s12974-022-02492-0. PMC9168645 35668399

[36] Wu Y. , Murray G. K. , Byrne E. M. , Sidorenko J. , Visscher P. M. , and Wray N. R. , GWAS of Peptic Ulcer Disease Implicates *Helicobacter pylori* Infection, Other Gastrointestinal Disorders and Depression, Nature Communications. (2021) 12, no. 1, 10.1038/s41467-021-21280-7, 1146. PMC7895976 33608531

[37] Nahar Z. , Monisha S. T. , Qusar M. S. , and Islam M. R. , Evaluation of Serum Interleukin-1 Receptor Antagonist Levels in Major Depressive Disorder: A Case-Control Study, Health Science Reports. (2023) 6, no. 4, 10.1002/hsr2.1175, e1175. PMC10050969 37008817

[38] Minbay M. , Khan A. , Ghasemi A. R. , Ingram K. K. , and Ay A. A. , Sex-Specific Associations Between Circadian-Related Genes and Depression in UK Biobank Participants Highlight Links to Glucose Metabolism, Inflammation and Neuroplasticity Pathways, Psychiatry Research. (2024) 337, 10.1016/j.psychres.2024.115948, 115948. 38788553

