# Supplementary material for: Plasma Proteomic Landscape of Multidimensional Sleep Health and Its Association With Depression Risk Under Genetic Susceptibility
Source: Depress Anxiety. 2026 Oct 9;2026:9191032. doi: 10.1155/da/9191032 (PMC13650738; doi:10.1155/da/9191032)
Supplement: Supplementary file 1 [file DA-2026-9191032-s001.docx]

***Supplementary Materials***

**Figure S1.** Flowchart of study participant selection in the UK Biobank.

**Legend:** Flow diagram illustrating the sequential exclusion process used to derive the final analytical cohort from the UK Biobank participants with available proteomics data (N = 52,998). Participants were excluded due to missing sleep-related variables (N = 9,704), non-White ancestry (N = 2,638), missing depression polygenic score data (N = 179), and baseline depression (N = 3,625). The final analytical sample included 36,852 participants after these exclusions.

**Figure S2**. Mediation analysis of the association between sleep pattern and incident depression through the proteomic signature.

**Legend:** Among individuals with intermediate sleep patterns, the indirect effect through the proteomic score was HR = 0.975 (95% CI, 0.962–0.988), while the direct effect was HR = 0.765 (95% CI, 0.635–0.921), with a proportion mediated of 8.55% (95% CI, 3.69%–27.92%). Among individuals with healthy sleep patterns, the indirect effect was HR = 0.929 (95% CI, 0.898–0.961), and the direct effect was HR = 0.592 (95% CI, 0.482–0.728), with a proportion mediated of 12.23% (95% CI, 6.63%–21.67%). The 95% confidence intervals for the proportions mediated were estimated using 1,000 nonparametric bootstrap resamples. Models were adjusted for age, sex, educational attainment, Townsend deprivation index, BMI, smoking status, alcohol status, moderate physical activity, cardiovascular disease, and diabetes.

**Figure S3**. Association between individual sleep components and risk of incident depression.

**Legend:** Multivariable Cox regression models were used to assess the associations between five individual sleep components and the risk of incident depression. The components include: sleep duration (normal: 7–8h; abnormal: < 7h or > 8h), chronotype (morning vs. evening person), insomnia (yes vs. no), snoring (yes vs. no), and daytime sleepiness (yes vs. no). All models were adjusted for age, sex, BMI, education level, smoking, drinking, physical activity, Townsend deprivation index, diabetes, and cardiovascular disease. Hazard ratios (HRs) and 95% confidence intervals (CIs) are presented, with the reference group indicated. Statistical significance was determined using two-sided p-values.

**Figure S4.** Sensitivity analysis excluding incident depression within the first year of follow-up.

**Legend:** Hazard ratios (HRs) and 95% confidence intervals (CIs) for the associations of sleep pattern, sleep proteomic signature score, and depression polygenic risk score (PRS) with incident depression, after excluding cases diagnosed within one year of baseline. Sleep pattern and sleep proteomic score were analyzed as categorical (low, intermediate, high) and continuous variables. PRS was analyzed as low (bottom 25%), intermediate (middle 50%), and high (top 25%) genetic-risk groups and as a continuous variable. Models were adjusted for age, sex, BMI, smoking, drinking, moderate physical activity, education, Townsend deprivation index, diabetes, and cardiovascular disease. This sensitivity analysis aims to mitigate the possibility of reverse causality.

**Table S1.** Sensitivity of elastic-net feature selection across alternative α values and λ-selection rules.

| **α** | **λ-selection rule** | **λ** | **Proteins selected, n** | **Primary 73 retained** | **Jaccard similarity** | **Score correlation** |
| --- | --- | --- | --- | --- | --- | --- |
| 0.10 | 1-SE rule (λ1SE) | 0.161258 | 91 | 73/73 (100.0%) | 0.802 | 0.99894 |
| 0.10 | Minimum CV error (λmin) | 0.063603 | 406 | 66/73 (90.4%) | 0.160 | 0.94529 |
| 0.25 | 1-SE rule (λ1SE) | 0.070792 | 76 | 73/73 (100.0%) | 0.961 | 0.99993 |
| 0.25 | Minimum CV error (λmin) | 0.025441 | 397 | 66/73 (90.4%) | 0.163 | 0.94200 |
| 0.50 | 1-SE rule (λ1SE) | 0.035396 | 73 | 73/73 (100.0%) | 1.000 | 1.00000 |
| 0.50 | Minimum CV error (λmin) | 0.012721 | 392 | 66/73 (90.4%) | 0.165 | 0.94076 |
| 0.75 | 1-SE rule (λ1SE) | 0.023597 | 72 | 72/73 (98.6%) | 0.986 | 0.99999 |
| 0.75 | Minimum CV error (λmin) | 0.008480 | 392 | 66/73 (90.4%) | 0.165 | 0.94033 |
| 1.00 | 1-SE rule (λ1SE) | 0.017698 | 71 | 71/73 (97.3%) | 0.973 | 0.99998 |
| 1.00 | Minimum CV error (λmin) | 0.006360 | 392 | 66/73 (90.4%) | 0.165 | 0.94011 |

**Note:** The primary elastic-net model used α = 0.50 and the 1-SE rule (λ1SE), selecting 73 proteins. Primary 73 retained denotes the number and percentage of proteins from the primary 73-protein set that were reselected under each alternative specification. Jaccard similarity quantifies overlap between the alternative and primary selected feature sets. Score correlation is the Pearson correlation between the alternative proteomic score and the primary proteomic score. λmin denotes the λ value yielding the minimum cross-validation error.

**Table S2.** Selection stability of the 73 primary sleep-related proteomic features across 50 repeated 10-fold cross-validation runs.

| **Protein** | **Primary coefficient** | **Selected runs** | **Selection frequency** |
| --- | --- | --- | --- |
| ACP5 | -0.011450 | 50/50 | 100% |
| ADAMTS8 | 0.015522 | 50/50 | 100% |
| ADM | -0.035321 | 50/50 | 100% |
| ALDH3A1 | -0.006361 | 50/50 | 100% |
| ALPP | -0.004655 | 50/50 | 100% |
| AMY2A | 0.014661 | 50/50 | 100% |
| APCS | -0.043008 | 50/50 | 100% |
| BCHE | -0.038402 | 50/50 | 100% |
| CA14 | 0.027506 | 50/50 | 100% |
| CCL16 | -0.007507 | 50/50 | 100% |
| CILP | -0.020580 | 50/50 | 100% |
| CPM | -0.011152 | 50/50 | 100% |
| DPP6 | 0.023847 | 50/50 | 100% |
| ERBB2 | -0.081433 | 50/50 | 100% |
| FABP4 | -0.023422 | 50/50 | 100% |
| GFRA1 | -0.035442 | 50/50 | 100% |
| GGT1 | -0.026427 | 50/50 | 100% |
| GPD1 | -0.006074 | 50/50 | 100% |
| GUSB | -0.001249 | 50/50 | 100% |
| HAVCR1 | -0.011322 | 50/50 | 100% |
| IGFBP1 | 0.007288 | 50/50 | 100% |
| IL1RN | -0.003846 | 50/50 | 100% |
| ITGAM | 0.083153 | 50/50 | 100% |
| LEP | -0.016833 | 50/50 | 100% |
| LRRN1 | 0.013305 | 50/50 | 100% |
| LTA4H | -0.008815 | 50/50 | 100% |
| MRC1 | -0.029266 | 50/50 | 100% |
| MXRA8 | 0.040484 | 50/50 | 100% |
| NTRK3 | 0.009689 | 50/50 | 100% |
| PAEP | 0.009336 | 50/50 | 100% |
| PLAT | -0.031125 | 50/50 | 100% |
| PRSS8 | -0.030624 | 50/50 | 100% |
| RBP5 | -0.012672 | 50/50 | 100% |
| RGMA | 0.031329 | 50/50 | 100% |
| SCLY | -0.006936 | 50/50 | 100% |
| SLITRK1 | 0.012608 | 50/50 | 100% |
| SSC5D | -0.010194 | 50/50 | 100% |
| TNFRSF6B | -0.005600 | 50/50 | 100% |
| WFIKKN2 | 0.036318 | 50/50 | 100% |
| ADAMTS13 | 0.024506 | 49/50 | 98% |
| CDON | -0.035550 | 49/50 | 98% |
| CNTN5 | 0.005516 | 49/50 | 98% |
| IGFBP3 | 0.024656 | 49/50 | 98% |
| LEFTY2 | 0.010003 | 49/50 | 98% |
| SPINK6 | -0.007269 | 49/50 | 98% |
| VIT | -0.008822 | 49/50 | 98% |
| BGLAP | -0.005840 | 47/50 | 94% |
| CHRDL2 | 0.007176 | 47/50 | 94% |
| CHGB | 0.009574 | 44/50 | 88% |
| CXADR | -0.005915 | 44/50 | 88% |
| CXCL17 | -0.002220 | 44/50 | 88% |
| FBP1 | -0.001782 | 44/50 | 88% |
| HEG1 | -0.003948 | 44/50 | 88% |
| VWA1 | -0.004943 | 44/50 | 88% |
| ADH4 | -0.001368 | 43/50 | 86% |
| DSG2 | 0.000817 | 43/50 | 86% |
| NCAN | 0.002387 | 43/50 | 86% |
| CCDC80 | -0.004026 | 36/50 | 72% |
| CD209 | 0.004853 | 36/50 | 72% |
| GGH | -0.001230 | 36/50 | 72% |
| INHBB | -0.002311 | 36/50 | 72% |
| SFRP4 | 0.011400 | 36/50 | 72% |
| ANGPTL1 | 0.004116 | 24/50 | 48% |
| CD83 | -0.001038 | 24/50 | 48% |
| CES1 | -0.000037 | 24/50 | 48% |
| CLMP | -0.005309 | 24/50 | 48% |
| EBI3_IL27 | 0.003623 | 24/50 | 48% |
| FUT3_FUT5 | -0.000620 | 24/50 | 48% |
| GAST | -0.000118 | 24/50 | 48% |
| IL1RAP | 0.000989 | 24/50 | 48% |
| LDLR | -0.000372 | 24/50 | 48% |
| STC1 | -0.000192 | 24/50 | 48% |
| TMPRSS11D | -0.000615 | 24/50 | 48% |

**Note:** The primary model used α = 0.50 and λ1SE. For each of 50 repetitions, 10-fold cross-validation was rerun using a different random fold assignment, followed by refitting the elastic-net model at the corresponding λ1SE. Across repetitions, the median number of selected proteins was 62 (IQR, 58–82), the median recovery of the primary 73-protein set was 84.9% (IQR, 78.1–100.0%), and the median Jaccard similarity with the primary set was 0.849 (IQR, 0.751–0.890). The median correlation between repeated-CV proteomic scores and the primary score was 0.999 (IQR, 0.998–0.999), and the median pairwise Jaccard similarity across repeated runs was 0.770 (IQR, 0.687–0.890). Overall, 48/73 proteins were selected in ≥90% of repetitions and 62/73 in ≥70% of repetitions; 11 proteins were selected in 48% of repetitions.

| **Table S3.** Definitions and coding of selected covariates used in the analysis. | | | |
| --- | --- | --- | --- |
| **Covariate** | **UK Biobank Field ID** | **Source variable** | **Coding/definition used in this study** |
| Educational attainment | 6138 | Qualifications | Categorized as college/university degree or above versus below college/university level. |
| Alcohol status | 20117 | Alcohol drinker status | Categorized as never, former, or current. |
| Moderate physical activity | 22038 | Moderate physical activity | Categorized as yes (≥150 min/week) or no (<150 min/week). |
| Cardiovascular disease (CVD) | 6150; 6153 (women); 6177 (men) | Vascular/heart problems diagnosed by doctor; medication use | Defined as physician-diagnosed heart attack, angina, stroke, or high blood pressure (Field 6150), or reported use of blood-pressure medication (Field 6153 for women and Field 6177 for men). |
| ***Note:*** CVD, cardiovascular disease. | | | |
